# Intravitreal ranibizumab monotherapy to treat retinopathy of prematurity zone II, stage 3 with plus disease

**DOI:** 10.1186/s12886-015-0001-7

**Published:** 2015-03-08

**Authors:** Marcel N Menke, Carsten Framme, Mathias Nelle, Markus R Berger, Veit Sturm, Sebastian Wolf

**Affiliations:** Department of Ophthalmology, Inselspital, Bern University Hospital, and University of Bern, Bern, Switzerland; University Eye Hospital, Medical School Hannover, Hannover, Germany; Department of Neonatology, Inselspital, Bern University Hospital, and University of Bern, Bern, Switzerland; Department of Ophthalmology, Cantonal Hospital St. Gallen, St. Gallen, Switzerland; Department of Ophthalmology, Cantonal Hospital Aarau, Aarau, Switzerland

**Keywords:** Retinopathy of prematurity, Ranibizumab, Bevacizumab, Plus disease

## Abstract

**Background:**

Treatment of retinopathy of prematurity (ROP) stage 3 plus with bevacizumab is still very controversial. We report the outcome of 6 eyes of 4 premature infants with ROP stage 3 plus disease treated with ranibizumab monotherapy.

**Methods:**

Six eyes of 4 premature infants with threshold ROP 3 plus disease in zone II, were treated with one intravitreal injection of 0.03 ml ranibizumab. No prior laser or other intravitreal therapy was done. Fundus examination was performed prior to the intervention and at each follow-up visit. Changes in various mean vital parameters one week post intervention compared to one week pre-intervention were assessed.

**Results:**

The gestational age (GA) of patient 1, 2, 3, and 4 at birth was 24 5/7, 24 5/7, 24 4/7, and 26 1/7 weeks, respectively. The birth weight was 500 grams, 450 grams, 665 grams, and 745 grams, respectively. The GA at the date of treatment ranged from 34 3/7 to 38 6/7 weeks. In one infant, upper air way infection was observed 2 days post injection of the second eye. Three eyes required paracentesis to reduce the intraocular pressure after injection and to restore central artery perfusion. After six months, all eyes showed complete retinal vascularisation without any signs of disease recurrence.

**Conclusions:**

Treatment of ROP 3 plus disease with intravitreal ranibizumab was effective in all cases and should be considered for treatment. One infant developed an upper air way infection suspicious for nasopharyngitis, which might be a possible side effect of ranibizumab. Another frequent complication was intraocular pressure rise after injection. More patients with longer follow-up duration are mandatory to confirm the safety and efficacy of this treatment.

**Trial registration number:**

NCT02164604; Date of registration: 13.06.2014

## Background

Retinopathy of prematurity (ROP) is a neovascular retinal disorder of premature born children, characterized by the development of retinal neovascularisation, macular dragging and eventually retinal detachment. ROP is a leading cause for childhood blindness, especially in developing countries [1]. Vascular endothelial growth factor (VEGF) plays an important role in the development of the disease. VEGF production is triggered by the avascular part of the retina and accumulation of VEGF eventually leads to neovascularisation and retinal detachment if not treated in time [2]. Currently, near-confluent laser therapy is recommended for treatment of the outer, avascular retina to destroy the cells that produce VEGF [3].

Recently, the BEAT ROP study tested the efficacy of intravitreal bevacizumab (Roche AG, Basel, Switzerland) for stage 3 plus ROP in a prospective, controlled, randomized, stratified, multicenter trial [4]. Authors found that bevacizumab showed a significant benefit for Zone I but not Zone II disease, with continuation of peripheral retinal vessel growths after treatment. The authors also concluded that safety could not be assessed due to the small sample size. Other authors raised concerns regarding the results of the BEAT ROP study [5]. Visual outcomes were not reported in the BEAT ROP trial. In addition, recurrence of ROP may have occurred after the study end point since late recurrence of ROP after bevacizumab treatment has been reported [4]. Also the dose used in the BEAT-ROP trial might have been too high [5].

Sato et al. could show that bevacizumab escapes from the vitreous into the general circulation and reduces systemic VEGF levels for weeks to months in premature infants [6]. In contrast, Carneiro et al. showed that ranibizumab does not alter systemic VEGF levels in adults [7]. Bearing this in mind together with a shorter half-life of ranibizumab in human nonvitrectomized eyes (7.15 days versus 9.82 days) we suspected a better safety profile for ranibizumab to treat stage 3 plus ROP [8]. The aim of this study was to demonstrate the effectiveness of intravitreal ranibizumab to treat ROP stage 3 plus disease. Here we present the outcome of 6 eyes.

## Methods

Six eyes of 4 children with confirmed ROP stage 3 plus in Zone II have been included. The diagnosis was confirmed by indirect ophthalmoscopy and scleral indentation. Parents were informed regarding the disease and treatment options. Laser-photocoagulation of the avascular retina was suggested. Alternatively, one intravitreal injection with 0.03 ml ranibizumab was offered for treatment. Parents decided for their children to receive intravitreal ranibizumab in these cases and signed an informed consent before the procedure. The Cantonal Ethics Committee of Berne was informed prior to treatment and approved therapy. No prior laser or other intravitreal therapy was performed. The injection procedure was planned in collaboration with the Department of Neonatology and was performed in a special intervention room at the neonatology unit. Patients were analgosedated, intubation was performed based on the decision of the neonatology team if the infant was unstable due to general clinical condition. All infants were continuously monitored during and after the procedure. Ranibizumab was injected with a 30-gauge needle through the conjunctiva approximately 1.5 mm behind the nasal limbus according to a procedure that was published previously using bevacizumab instead of ranibizumab [9]. Disinfection of the injection side was done with polyhexamethylene biguamide (Polyhexanide, based on Lavasept® solution, B Braun Melsungen AG, Melsungen, Germany) eyedrops. Skin disinfection was done with Octenidindihydro-chlorid (Octenisept, Steinberg Pharma AG, Winterthur, Switzerland). Both agents were used to avoid iodine absorption which could be problematic in premature infants. The eye was stabilized with a toothed forceps while the dose of 0.03 ml ranibizumab was injected. After injection, the intraocular pressure was assessed by bulbus palpation. Central artery perfusion was checked by indirect ophthalmoscopy immediately after injection. If necessary, paracentesis was performed to release pressure after intravitreal injection. Photographic documentation was done with a HEINE Video OMEGA® 2C indirect ophthalmoscope (Heine Optotechnik, Herrsching, Germany) An ophthalmic antibiotic eye drop (Tobrex eye drops; 3 mg Tobramycin/1 ml, Alcon Grieshaber AG, Schaffhausen, Switzerland) was prescribed for the treated eye to begin immediately and be continued 4 times a day for 3 days. All patients were re-examined the following day, three days after treatment, one week after treatment, and then depending on regression of ROP and status of vascularisation of the avascular retina. In addition, patients were continuously monitored in the neonatology unit for possible side effects of the treatment.

## Results

Female patient 1 had a gestational age (GA) of 24 5/7 weeks at birth and weight 500 grams. At the left eye severe ROP 3 plus in zone II over 7 clock hours was diagnosed at gestational week 32 6/7. OS was injected 24 hours after diagnosis. OD was observed having ROP 3 without plus disease in zone II. One week post treatment plus disease showed clear regression in the left eye. Unfortunately, 14 days after treating OS, the right eye progressed to ROP 3 plus disease over 6 clock hours (gestational age 37 3/7) and therefore an injection was performed the same day of diagnosis. Immediately after the injection procedure anterior chamber paracentesis was required due to high intraocular pressure with poor central artery perfusion observed with indirect ophthalmology. Three days after injecting OD the patient developed moderate upper airway infection suspicious for nasopharyngitis that was tested positive for Klebsiella pneumonia and successfully treated with antibiotics. One week past treatment the neovascularisations showed clear regression and plus disease was absent in both eyes. Three months past the injection in OS, vascularisation of the peripheral retina was complete in OD but still incomplete in OS. 6 months after treatment vascularisation was complete in both eyes without recurrence of ROP.

**Figure 1:**
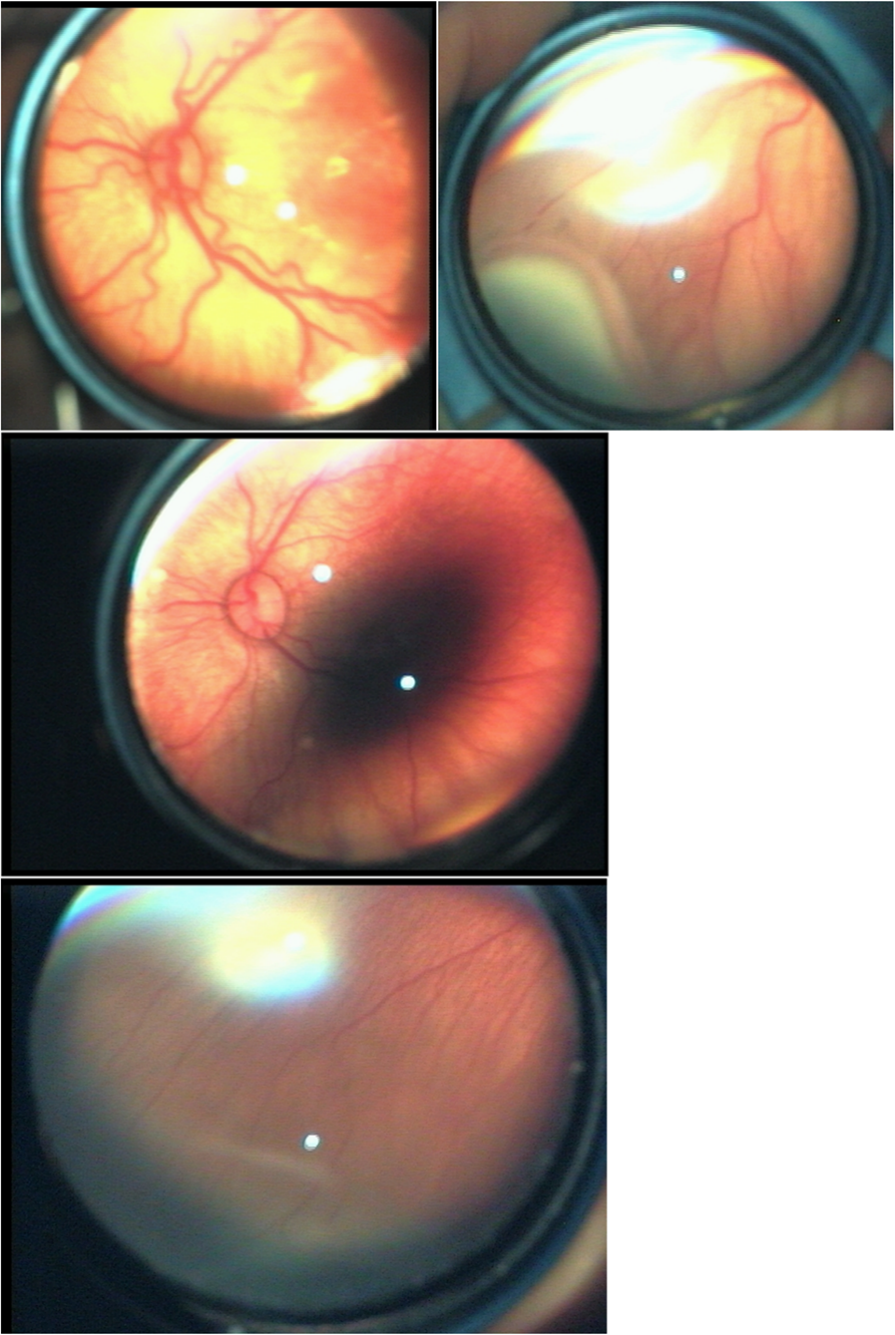
**Funduspictures taken from patient 4.** Top: Funduscopic findings before ranibizumab injections show plus disease with tortuous, dilated blood vessels. On the right, the ridge with neovascularisations can be appreciated under sclera indentation. Middle: One week post-injection plus disease has almost completely disappeared. Bottom: Six month post treatment, retinal vascularisation is complete without recurrence of ROP.

Patient 2 is the twin sister of patient 1 with a birth weight of 450 grams. Her right eye required treatment at GA of 34 3/7 weeks for severe ROP 3 plus disease in zone II over 8 clock hours. After injection, anterior paracentesis was required to reduce pressure since central artery perfusion was poor directly after injection. One week post treatment plus disease was absent. At 3 and 6 months follow up, vascularisation was complete with no recurrence of ROP. Figure 1 shows fundus findings before injection, and 1 week and 6 months after treatment.

Female patient 3 had a GA of 24 4/7 weeks at birth and weight 665 grams. The right eye received treatment for ROP 3 over 6 clock hours with plus disease in Zone II at GA of 34 4/7 weeks without any occurring complication. The left eye also showed ROP 3 over 6 clock hours with plus disease in Zone II and required treatment at GA 35 5/7 weeks. Also anterior chamber paracentesis was required due to high intraocular pressure in OS. One week after intervention, plus disease has disappeared in both eyes. At 3 months follow up, vascularisation was incomplete in OD with ROP 1 without plus disease in Zone II. Vascularisation of OS was complete. At 6 months endpoint, vascularisation was complete in both eyes without recurrence of ROP.

Female patient 4 had a GA of 26 1/7 weeks at birth and weight 745 grams. The left eye showed ROP 3 over 9 clock hours in Zone II with plus disease and required treatment at GA of 38 6/7 weeks without any occurring complication. Complete regression of plus disease was seen 1 week post injection. At 3 and 6 months follow up, vascularisation was complete with no recurrence of ROP.

Table 1 shows baseline data of all patients and concomitant diseases. Main vital parameters such as mean systolic and diastolic blood pressure, mean pulse rate, and required oxygen therapy (mean FiO2) have been monitored. In addition, patients were monitored for dips of oxygen saturation and lowest values were recorded for each day. Vital parameters one week before treatment were compared with values 1 week after injection. Findings are presented in Table 2. Minimal oxygen saturation levels (dips in O^2^ saturation as a sign for respiratory stability) were lower 1 week before the treatment (mean lowest O^2^ saturation pre-treatment was 61.89 ± 2.63%) compared to 1 week after treatment (mean lowest O^2^ saturation post-treatment was 70.54 ± 1.12%; p = 0.0037). All other vital parameters in exception of respiratory stability, showed no significant difference after treatment with ranibizumab.

**Table 1:** **Baseline characteristics and concomitant diseases**

**Patient**	**GA at birth**	**AA at injection**	**Birthweight**	**Comcomitant diseases**
1	24 5/7	OD: 37 3/7	500 g	apnea and bradycardia syndrome, persisten ductus arteriosus, RDS, pneumonia, kidney insufficiency, arterial hypotension, hyperbilirubinemia, anemia
		OS: 32 6/7		
2	24 5/7	OD 34 3/7	450 g	apnea and bradycardia syndrome, persisten ductus arteriosus, RDS, pneumonia, arterial hypotension, hyperbilirubinemia, anemia, hernia inguinalis, sepsis, neutropenia
3	24 4/7	OD: 34 4/7	665 g	apnea and bradycardia syndrome, , RDS, neonatal infection, urinary tract infection, intracerebral hemorrhage pneumonia, arterial hypotension, hyperbilirubinemia, anemia, bronchopulmonary dysplasia, kidney insufficiency
		OS: 35 5/7		
4	26 1/7	OS: 38 6/7	745 g	apnea and bradycardia syndrome, RDS, hyperbilirubinemia, anemia

GA = gestational age; RDS = respiratory distress syndrome; AA = Adjusted age.

**Table 2:** **Vital parameters one week before and after ranibizumab injection**

	**1 week pre-injection**	**1 week post-injection**	**p-value**
**Mean systolic blood pressure**	61.07 ± 1.5 mmHg	64.39 ± 1.1 mmHg	ns
**Mean diastolic blood pressure**	31.43 ± 1.1 mmHg	32.61 ± 1.2 mmHg	ns
**Mean heart rate**	154.9 ± 1.4	153.9 ± 1.9	ns
**Mean respiratory frequency**	54.46 ± 0.9	52.46 ± 1.1	ns
**Mean FiO**_**2**_	21.89 ± 1.8%	22.39 ± 2.1%	ns
**mean lowest O**_**2**_ **saturation**	61.89 ± 2.6%	70.54 ± 1.1%	0.0037

ns = not significant.

## Discussion

Intravitreal bevacizumab has become increasingly popular to treat ROP 3 plus disease and zone one disease. In 2007 first case reports were published, showing fast regression of ROP after single injections of the drug either as single line treatment or in combination with laser [10,11]. The observation of fast ROP regression and complete retinal vascularisation after anti-VEGF injection led to an increasing popularity of this treatment option. Multiple reviews have been published discussing the use of bevacizumab in ROP even before the first randomized prospective study data of the BEAT ROP trial became available [12-14]. The BEAT ROP trial showed favorable results of bevacizumab in Zone I but not in Zone II disease compared to laser therapy [4]. Although it was the first prospective, randomized trial, the results raised several concerns. Study population was too small to assess safety of bevacizumab treatment. The mortality of children was 6.6% in the bevacizumab treated group vs. 2.6% in the laser group. Although the difference was not statistically significant, mortality needs to be closely monitored in these patients [5]. In addition, little is known regarding the functional outcome of eyes treated with intravitreal bevacizumab.

These concerns led us to the idea of treating severe ROP with intravitreal ranibizumab. Ranibizumab seems to have a shorter half-life in human non-vitrectomized eyes (7.15 days versus 9.82 days of bevacizumab) which would be favorable in premature infants were we want the lowest systemic concentration for the shortest possible duration [8]. It has been shown, that ranibizumab does not alter systemic VEGF levels in adults [7]. However, recently Hoerster and colleagues testes serum VEGF levels in a premature infant after one injection of ranibizumab to treat ROP 3 plus in zone I. They found suppressed systemic VEGF levels below detection limit for about 2 weeks post-injection. This suppression might be explained by an impaired blood-retina barrier of the immature retina at this age, as ranibizumab does not seem to change systemic VEGF levels in adults. Hoerster et al. found that four weeks after bilateral treatment, systemic VEGF levels returned to normal values [15]. In a case series of 11 eyes treated with bevacizumab, systemic VEGF levels were suppressed for at least 7 weeks post-injection. In addition, Sato et al. measured serum VEGF level in infants after intravitreal bevacizumab injection and found systemic reduction of VEGF levels for at least 2 weeks after injection [6]. This data points in the direction of a better safety profile for ranibizumab [16]. One can assume that ranibizumab shows less systemic drug activity after intravitreal injection and this might be a possible explanation why 2 of our patients required bilateral injections within a short period of few weeks. With bevacizumab, improvement of the untreated partner eye has been reported in a case of an 8 year-old girl with uveitic cystic macular edema indicating that the systemic drug action might even be strong enough to “treat” the partner eye at least in older children or adults [17].

In our study group all eyes showed rapid regression of ROP after single injection of 0.3 mg ranibizumab. No late recurrence of ROP was seen over a period of 6 months and all patients finally gained complete retinal vascularization. The treatment procedure appears to be safe but short-term IOP increase is frequent (50% of our cases). In all cases of increased IOP, indirect ophthalmoscopy after injection revealed poor central artery perfusion which let us performing paracentesis to reduce pressure. After this procedure, central artery perfusion improved immediately. To our knowledge, no studies investigated IOP increase after anti-VEGF injection in infants. However, transient or even sustained IOP elevation after anti-VEGF injection in adult patients is common knowledge and well described [18-20].

The injected volume plays an important role in this IOP matter. In our cases 0.03 ml (0.3 mg) ranibizumab was injected. There is still ongoing discussion, how much anti-VEGF drug is required to treat ROP sufficiently with one single injection. Sears et al. estimated that approximately 0.5-1.0 mg of bevacizumab is 10000 times the concentration needed to neutralize the highest measured concentration of VEGF. [21] Other reports have shown that lower doses of bevacizumab (0.375 g) achieved regression of ROP changes [22]. The dose used in the BEAT-ROP trial was half the adult dose. However, the vitreous volume of an infant at 34 weeks is less (1.6 ml vs. 4.0 ml), as is the retinal surface area (450 mm^2^ vs. 1240 mm^2^). In addition, the body weight is about one-fiftieth of an adult person [5]. Most likely the ranibizumab dose used in our cases was unnecessarily high but currently there is no valid dosage data available and the risk of under-treatment should be avoided since intravitreal injections are invasive procedures that should not be repeated if not necessary.

Few other reports have been published using ranibizumab for ROP treatment. Lin C.J. et al. report a case of an extreme low-birth-weight infant that was previously unsuccessfully treated with intravitreal bevacizumab and laser for aggressive zone I disease. After one injection of 0.25 mg ranibizumab, ROP disappeared completely and the retina developed complete vascularisation [23]. The same group published a series of cases of ROP 3 treated with ranibizumab and showed good response to treatment with no short-term systemic side effects or major ocular side effects [24]. Castellanos M.A. et al. presented a case series of six eyes treated with one injection of ranibizumab and followed patients over 3 years. All patients gained complete retinal vascularisation with full regression of ROP. After 3 years, average visual acuity (snellen equivalent) was 20/30, which implies normal ocular growth and function after ranibizumab treatment in these cases [25]. Mota A. et al. used ranibizumab in combination with laser to successfully treat zone I disease in 2 cases. No systemic or ocular adverse effects have been observed [26].

In our case series, upper airway infection suspicious for nasopharyngitis has been observed in one patient after injection as a possible side effect of ranibizumab treatment. Nasopharyngitis has been observed in 12.5 to 16.4% of age-related macular degeneration cases that were treated with ranibizumab in three phase III studies [27-29]. Rather trivial in adult patients, nasopharyngitis might be a severe complication in premature born children with pre-existing breathing problems due to a respiratory distress syndrome.

In addition, several vital parameters have been observed one week before and after treatment. No significant change of pulse rate, respiratory rate, systolic and diastolic blood pressure and required mean FiO^2^ was found in our patient group. Only minimal oxygen saturation levels (dips in O^2^ saturation) were significantly lower 1 week before the treatment (mean lowest O^2^ saturation pre-treatment was 61.89 ± 2.63%) compared to 1 week after treatment (mean lowest O^2^ saturation post-treatment was 70.54 ± 1.12%; p = 0.0037). These findings are most likely related to the fact that infants simply become older and that their respiratory distress syndrome or bronchopulmonary dysplasia further improved.

There is the limitation that only 4 patients have been treated. Beside the small sample size, this case series has further limitations. The observation period has only been 6 months. No visual acuity values have been reported. In addition, no patients with zone 1 disease have been included in this case series. This was the group that showed most benefit in the BEAT-ROP trial. Further prospective studies with larger study populations are required to evaluate the effectiveness and safety of ranibizumab for ROP treatment. Our case series has not the power to assess safety of ranibizumab treatment. Funduscopy showed that all patients developed complete retinal vascularisation after treatment, but since no fluorescein angiography was performed, it remains unknown if vascularisation was normal or abnormal. Based on the observations of the BEAT-ROP trial and our own observations, follow up should be at least every 2–3 weeks until vascularization is complete, approximately 20+ weeks following the last antiVEGF injection.

## Conclusions

In conclusion, one single injection of ranibizumab was effective in treating ROP 3 plus disease in zone 2 in all cases without showing late recurrence of ROP. Treatment led to complete retinal vascularisation within the follow up period of 6 months. Preterm infants treated with intravitreal ranibizumab might have better outcome than preterm infants receiving conventional laser therapy since peripheral retinal destruction was suggested as the primary cause of visual field loss and development of high myopia [30]. There was no significant negative effect on basic vital parameters related directly to the treatment. Nasopharyngitis has been observed in one case as a possible side effect of treatment as well as frequent post-injection increase of intraocular pressure. Further prospective studies with larger study population are required to evaluate the effectiveness and safety of ranibizumab for ROP treatment.

### Ethics approval

IRB (Cantonal Ethics Committee of Berne, Switzerland) approval for this work was obtained.
